# Redescription of *Heligmosomoides neopolygyrus*, Asakawa and Ohbayashi, 1986 (Nematoda: Heligmosomidae) from a Chinese Rodent, *Apodemus peninsulae* (Rodentia: Muridae); with comments on *Heligmosomoides polygyrus polygyrus* (Dujardin, 1845) and related species in China and Japan

**DOI:** 10.1051/parasite/2012194367

**Published:** 2012-11-15

**Authors:** J. Massoni, M.C. Durette-Desset, J.P. Quéré, F. Audebert

**Affiliations:** 1 Université Pierre et Marie Curie, Paris, UMR 7207 CNRS-MNHNUPMC “Centre de recherche sur la Paléobiodiversité et les Paléoenvironnements – CR2P”. MNHN 57, rue Cuvier CC 48 75231 Paris Cedex 05 France; 2 Université Paris-Sud, UMR 8079 CNRS-UPSUD Écologie Systématique et Évolution, Équipe Biodiversité, Systématique et Évolution Bâtiment 360 91405 Orsay Cedex France; 3 Département de Systématique et Évolution, Muséum national d’Histoire naturelle, UMR 7138 CNRS-MNHN-UPMC 57, rue Cuvier CC 52 75231 Paris Cedex 05 France; 4 INRA, UMR 1062 CBGP, Campus international de Baillarguet, CS 30016 34988 Montferrier-sur-Lez Cedex France

**Keywords:** *Heligmosomoides* spp., Nematoda, Trichostrongylina, Heligmosomoidea, Rodent, Muridae, China, Japan, taxonomy, *Heligmosomoides* spp., Nematoda, Trichostrongylina, Heligmosomoidea, Rongeur, Muridae, Chine, Japon, taxinomie

## Abstract

*Heligmosomoides neopolygyrus*, Asakawa and Ohbayashi, 1986 (Nematoda, Heligmosomoidea) is redescribed from *Apodemus peninsulae* from Rangtang, Sichuan, China. A morphological review of the *Heligmosomoides* spp. belonging to the “*polygyrus* line” proposed by Asakawa (1988) is made using new characters. This enabled us to distinguish two subspecies in *Mus musculus* (*Heligmosomoides polygyrus bakeri* from Japan and *H. p. polygyrus* from China) and two valid species in *Apodemus* spp. (*H. neopolygyrus* from Japan [in *A. peninsulae*] and from China [in *A. agrarius*] and *H. asakawae* from China [in *A. uralensis*]). Three parasite species of *A. agrarius* and *A. peninsulae*, previously identified by Asakawa *et al.* (1993) as *H. neopolygyrus*, are considered to be *Heligmosomoides incertae sedis*. This is the first report of *H. neopolygyrus* in *A. peninsulae* from China.

## Abstract (fr)

*Heligmosomoides neopolygyrus*, Asakawa et Ohbayashi, 1986 (Nematoda : Heligmosomoidea) parasite d’*Apodemus agrarius* au Japon est redécrit chez un *A. peninsulae* originaire de Chine. Une revue des espèces du genre *Heligmosomoides* spp. appartenant à la lignée “*polygyrus*” caractérisée par Asakawa (1988) est faite en utilisant de nouveaux caractères. Ceci nous a permis de confirmer la distinction de deux sous-espèces chez *Mus musculus* (*Heligmosomoides polygyrus bakeri* au Japon et *H. p. polygyrus* en Chine) et de deux espèces valides chez *Apodemus* spp. [*H. neopolygyrus* au Japon (chez *A. peninsulae*) et en Chine (chez *A. agrarius*) et *H. asakawae* en Chine (chez *A. uralensis*)]. Trois espèces parasites d’*A. agrarius* et d’*A. peninsulae* qui ont été identifiées par Asakawa *et al.* (1993) comme *H. neopolygyrus* sont considérées comme des *Heligmosomoides incertae sedis*. C’est la première fois qu’*H. neopolygyrus* est signalé chez un *A. peninsulae* en Chine.

## Introduction 

The genus *Heligmosomoides* Hall, 1916 (Heligmosomidae) is widespread in the Holarctic region and is found mainly in the Arvicolinae but also in the Murinae. Asakawa (1988) divided the species of the genus into five categories namely the “*travassosi-douglasi* line”, the “relic group”, the “*laeviscarolinensis* line”, the “*longicirratum-longispiculatus* line” and the “*polygyrus* line”. The last line parasitizes only *Mus* and *Apodemus*. Until now, three species belonging to this line have been described or recorded from China: *Heligmosomoides polygyrus polygyrus* (Dujardin, 1845); *H. neopolygyrus* Asakawa and Ohbayashi, 1986 and *H. asakawae* Tenora and Barus, 2001, (Asakawa *et al.*, 1990, 1992, 1993). *H. neopolygyrus* and *H. p. bakeri* Durette-Desset *et al.*, 1972 are present in Japan (Asakawa & Ohbayashi, 1986; Hasegawa *et al.*, 1983).

In this study we redescribe *H. neopolygyrus* from Sichuan Province (central China) in *Apodemus peninsulae* (Muridae). The use of new morphological characters on the present and previously published material allows us to examine the validity of some reports of the “*polygyrus* line” described as *H. neopolygyrus*, as well as the distribution of the genus *Heligmosomoides* in Chinese and Japanese Muridae.

## Materials and Methods

Rodent hosts were collected in June 2004, as part of a French-British-Chinese program, for which the main goals were the screening of human populations for alveolar echinococcosis and the study of its transmission. The study area was located in Rangtang, Sichuan, China. The rodents were weighed and dissected in the field to determine the sex and reproductive status. Heads and tissue samples (or the whole body for a few specimens) were preserved for identification (Courant *et al.*, 1999). The nomenclature of the rodents follows Wilson & Reeder (2005).

The material studied here came from a single specimen of *A. peninsulae* (Thomas, 1907). The small intestine was preserved in 5 % formalin and transported to the Museum national d’Histoire naturelle (MNHN) in Paris, France, one month after collection. It was then transferred to 70 % ethanol. To determine the precise intestinal location of the parasites, the small intestine (SI) was divided longitudinally into four equivalent sections (SI 1 to SI 4) numbered from the pylorus to the caecum. Nematodes were collected from each section and stored in 70 % ethanol. They were examined as temporary mounts in lactophenol. The synlophe was studied following the method of Durette-Desset (1985) and the axis of orientation following that of Durette-Desset & Digiani (2005). The total number of cuticular ridges reported, is followed by the number of dorsal ridges and the number of ventral ridges in parentheses. The ridges were numbered from left to right, from 1 to n on the dorsal side, and from 1’ to n’ on the ventral side. The nomenclature used for the study of the caudal bursa follows Durette-Desset & Chabaud (1981) and Durette-Desset & Digiani (2012). The nomenclature for the parasites used above the family group follows Durette-Desset & Chabaud (1993). Measurements are given in micrometers, unless otherwise stated. Specimens studied have been deposited in the Helminthological Collection of the MNHN.

## Results

### Heligmosomoides Neopolygyrus Asakawa and Ohbayashi, 1986

Material: 88 females and 84 males, MNHN 442MQ. Material studied: 13 males and ten females

Host: *Apodemus peninsulae* (Thomas, 1907) (Rodentia: Muridae: Murinae).

Site in host: all specimens were found in SI 1.

Geographic origin: Rangtang, Sichuan, China. J.P. Coll.: Quéré, June 2004.

#### • Redescription (Figs 1-16)

Small nematode coiled along ventral side having two to four sinistral spirals in males and four to eight in females. Deirids setiform, situated at level of excretory pore (Fig. 4), observed in one male and one female.

**Figs 1–16.**
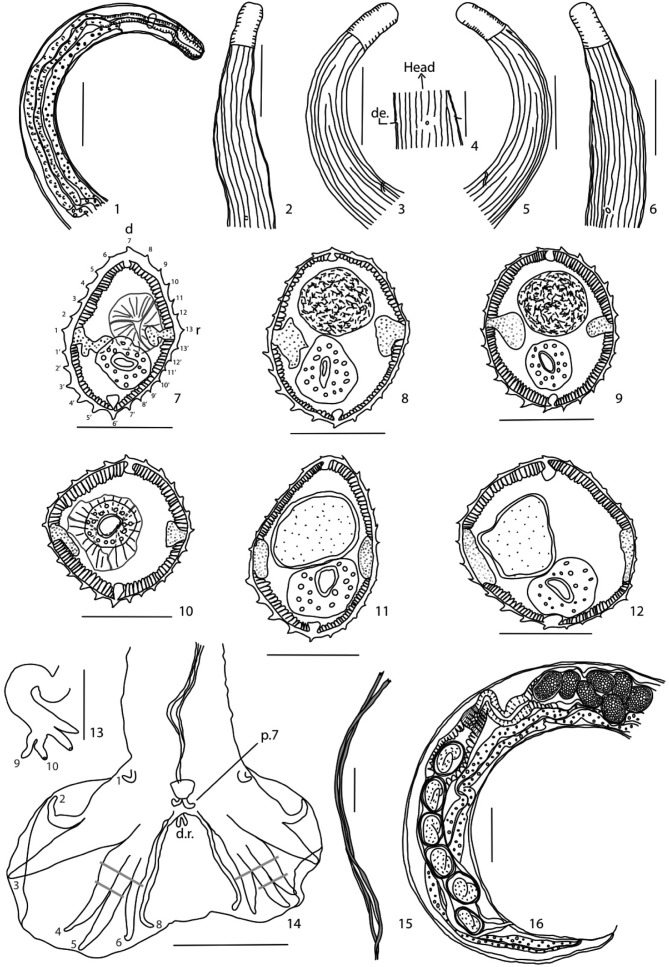
*Heligmosomoides neopolygyrus* Asakawa and Ohbayashi, 1986, in *Apodemus peninsulae*, from China: 1-6, male, anterior extremity, 1, right lateral view, 2-3, origin of cuticular ridges, 2, dorsal view, 3, right lateral view, 4, detail of excretory pore and deirids, ventral view, 5-6, origin of cuticular ridges, 5, left lateral view, 6, sub-ventral view; 7-12, transverse sections of body, 7-9, male, 7, at level of esophago-intestinal junction, 8, at mid-body, 9, within distal fifth, 10-12, female, 10, at level of esophago-intestinal junction, 11, at midbody, 12, within distal fifth; 13-15, male, 13, dorsal ray with rays 9 and 10, ventral view, 14, caudal bursa, ventral view, 15, spicules, *in situ*, ventral view; 16, female, posterior extremity, right lateral view. Scale bar: Figs 1-3, 5-6, 14, 16: 100 μm. Figs 4, 7-12, 15: 50 μm. Fig. 13: 20 μm. Abbreviations: de: deirids, r: right side, d: dorsal side, d.r.: dorsal ray, p.7: papillae 7. Transverse sections are oriented and numbered as in Fig. 7.

Small nematode coiled along ventral side having two to four sinistral spirals in males and four to eight in females. Deirids setiform, situated at level of excretory pore (Fig. 4), observed in one male and one female. Synlophe (studied in seven males and three females): in both sexes the cuticle bears longitudinal, continuous ridges without struts. Five ridges appear posterior to cephalic vesicle (Figs 2, 3, 5, 6), other ridges appear at different levels between cephalic vesicle and excretory pore; roughly equivalent numbers on dorsal and ventral sides (Figs 3, 5). Ridges disappear at about 130 anterior to caudal bursa in male, 110 anterior to caudal extremity in females. Number of ridges: at level of esophago-intestinal junction; 23, 26 (13D/13V see Fig. 7) in two males, 25, 26 (13D/13V, see Fig. 10) in two females; at mid-body, 24-27 (13D/13V, see Fig. 8) in seven males, 24-26 (13D/11V, see Fig. 11) in three females; within distal fifth, 25-27 (12D/14V, see Fig. 9) in three males, 25-31 (13D/14V, see Fig. 12) in three females. Left ventral ridges slightly larger than the other ridges. Axis of orientation sub-frontal directed from right to left (Figs 7-12).

Male: caudal bursa dissymmetrical with right lobe larger than left lobe (Fig. 14). Prebursal papillae well developed (Fig. 14). Caudal bursa pattern of type 2-3, for both lobes. Rays 3 thicker and longer than rays 2. In both lobes, rays 6, arising first from common trunk of rays 4-6. Rays 6 parallel to rays 8 and very close to them (Fig. 14). Rays 8, of similar length, arising at base of common trunk of rays 2-6. Extremities of rays 8 curved dorsally. Dorsal ray very small divided within distal third into two branches, each branch divided into two twigs, rays 9 (external branches) slightly shorter than rays 10 (internal branches) (Fig. 13). Rays 9 and 10 arising at same level as division of dorsal ray. Spicules poorly sclerotised, subequal, ending in sharp tip (Fig. 15). Gubernaculum absent. Genital cone, bearing two long papillae 7 on dorsal lip (Fig. 14). Papilla zero not observed. Measurements of 13 males are listed in Table 1.

**Table I.** Comparison between measurements (µm) of *Heligmosomoides neopolygyrus* from *Apodemuspeninsulae* in Japan (Asakawa & Ohbayashi, 1986) and from China (this article).

			Asakawa and Ohbayashi, 1986	This article
			Min-Max	Min-Max
Male		Number of specimens observed	5	13
		Total length	7,60–10,000	4,625–6,400
		Maximum width	150–200	80–100
		Length of cephalic vesicle	54–83	40–65
		Width of cephalic vesicle	37–56	30–40
		Distance from nerve ring to anterior extremity	188–223	130–170
		Distance from deirids to anterior extremity	not observed	210
		Distance from excretory pore to anterior extremity	254–464	195–390
		Length of esophagus	557–636	465–650
		Ratio esophagus / total length	6 to 7 %	7 to 12 %
		Length of right spicule	510–640	370–620
		Length of left spicule	510–640	370–620
		Ratio spicule length/ Total length	6 to 7 %	7 to 12 %
		Gubernaculum	no data	absent
		
		Number of ridges at midbody	28–35	24–27

Female		Number of specimens observed	5	10
		Total length	17,500–27,300	9,600–11,500
		Maximum width	150–320	70–150
		Length of cephalic vesicle	51–76	45–60
		Width of cephalic vesicle	48–60	35–40
		Distance from nerve ring to anterior extremity	127–191	115–170
		Distance from deirids to anterior extremity	n.o.	230
		Distance from excretory pore to anterior extremity	239–347	210–300
		Length of esophagus	378–576	530–685
		Ratio esophagus / Total length	2 %	3 to 7 %
		Length of caudal tip	no data	3–20
		Length of vagina vera	no data	12–25
		Length of tail	108–118	70–115
		Distance from vulvar opening to tail	315–410	230–330

	General Branch	Length of vestibule	no data	385–570
		Length of sphincter	no data	40–60
		Width of sphincter	no data	30–50
		Length of infundibulum	no data	130–240
		Length of uterine branch	no data	1,170–2,370
		Ratio uterus/ Total length	no data	10 to 22 %

	Eggs	Number of eggs	no data	8–45
		Length of eggs	76–87	50–75
		Width of eggs	54–60	30–50

		Number of ridges at midbody	29–33	25–26

Female: monodelphic. Vestibule very long. Tail rounded with caudal spine (Fig. 16). Measurements of ten females are listed in Table I.

#### • Differential diagnosis

The specimens described above belong to the genus *Heligmosomoides* Hall, 1916 (Heligmosomoidea: Heligmosomidae), as redefined by Durette-Desset (1968), which is characterized mainly by longitudinal cuticular ridges, a poorly developed dorsal ray, a very long vestibule and long deirids. Asakawa (1988) divided the genus into five categories one of which was the “*polygyrus* line”. This line was proposed for *Heligmosomoides* parasitic only in Muridae (*Mus* and *Apodemus*) and is made up of *H. neopolygyrus* Asakawa and Ohbayashi, 1986 in *A. peninsulae* from Japan and three subspecies of *Heligmosomoides polygyrus*: *H. p. polygyrus* (Dujardin, 1845) in *Apodemus* spp. and rarely in *Mus musculus* from the Palearctic region (Eurasia, Japan); *H. p. corsicus* Durette-Desset, 1968, in *M. musculus* from Corsica; and *H. p. bakeri* Durette-Desset *et al.*, 1972, in *M. musculus* from North America and from Japan (Hasegawa *et al.*, 1983; Yokoyama *et al.*, 1985). The “*polygyrus* line” was differentiated from the other lines by very short spicules, small ridges, without a prominent size gradient and very narrow intervals between the ridges (Asakawa, 1988). In this line, *H. neopolygyrus* is distinguished only by the absence of a swelling at the base of the externo-dorsal rays (rays 8) (Asakawa & Ohbayashi, 1986). Tenora & Barus (2001) raised the three subspecies of *H. p. polygyrus*, *H. p. bakeri* and *H. p. corsicus* to the species level. Cable *et al.* (2006) confirmed the specific status of *H. polygyrus* and *H. bakeri* using internal transcribed spacer (ITS) but Maizels *et al.* (2011) contested this result using another gene (CO1). Thus, the taxonomic ranks of *H. polygyrus* and *H. bakeri* are still the focus of discussion and in this article we follow the morphological study of Durette-Desset *et al.* (1972) are performed until further molecular studies on this subject.

Tenora & Barus (2001) considered the specimens described by Asakawa *et al.* (1992) as *H. p. polygyrus* in *Apodemus uralensis* (= *A. microps*) from China (Ulumuchi = Urumqi) to represent a new species and named it *H. asakawae*. They differentiated it from *H. p. polygyrus* by only one character, the “morphology of medio-dorsal ray”. Tenora *et al.* (2003) wrote “the dorsal rib of male differs distinctly morphologically from that of the species *H. p. polygyrus*”. From the drawing of Asakawa *et al.* (1992), we interpreted this character as the relative length of rays 9 and 10. In *H. p. polygyrus*, rays 9 are markedly shorter than rays 10, in *H. asakawae*, left ray 9 is as long as left ray 10 and right ray 9 is longer than right ray 10 (Table II).

**Table II.** Morphological characters of which the four in columns 3 (a, b), 4, 5, 6 differentiate the five species of *Heligmosomoides* belonging to the “*polygyrus* line” defined by Asakawa, 1988.

		This article				
	Asakawa & Ohbayashi (1986)	Arisal of rays 6 compared to the arisal of rays 4 and 5 on their common trunk				
Species	Swelling on 8 rays	Right lobe	Left lobe	Rays 6 // to rays 8	Direction of extremities of rays 6	Comparative length of rays 9 and 10
*Heligmosomoides p. polygyrus*	present	proximally	proximally or at same level	absent	curved to dorsal ray	9 shorter 10
*Heligmosomoides p. bakeri*	present	proximally	proximally	absent	curved to dorsal ray	9 shorter10
*Heligmosomoides p. corsicus*	present	proximally or at same level	proximally	absent	curved to dorsal ray	9 shorter10
*Heligmosomoides asakawae*	present	distally	distally	absent	curved to rays 5	left 9 as similar size as 10; right 9 longer than ray 10
*Heligmosomoides neopolygyrus*	absent	proximally	proximally	present	curved to rays 5	9 shorter than 10 or 9 as similar size as 10

The Chinese specimens have the characters of the “*polygyrus* line” and shared along with *H. neopolygyrus* the absence of a swelling at the base of the externo-dorsal ray. However, using only one character, the relative lengths of rays 9 and 10 for *H. asakawae* and the absence of the swelling at the base of rays 8 for *H. neopolygyrus*, to distinguish these species from *H. polygyrus sensu lato* seems to us insufficient to assure of their validity as species.

Using characters of the caudal bursa, the three subspecies of *H. polygyrus* share the same characters, whereas *H. asakawae* and *H. neopolygyrus* each have at least two other specific characters. In *H. asakawae*, rays 6 arise distally to the level of the divergence of rays 4 and 5. In *H. neopolygyrus*, rays 6 and rays 8 are close together and parallel. In addition, in *H. asakawae* and *H. neopolygyrus* the extremities of rays 6 are curved towards the extremities of rays 5 whereas in the three subspecies of *H. polygyrus* they are curved towards the dorsal ray (Table II).

The specimens described above have all the characters of *H. neopolygyrus* (Table II), but they can be differentiated by several elements from the type material described from Japan. Our specimens are about one third smaller, with smaller spicules but the spicule length/ body length ratio is larger (7–12 % *versus* 6–7 %); the ventral cuticular ridges are slightly larger than the dorsal ones; the number of ridges at midbody in the males is 24–27 *versus* 28–35 and in the females is 24–26 *versus* 29–33 (Table I).

Durette-Desset (1968) and Durette-Desset *et al.* (1972) demonstrated that in *H. polygyrus*, a differentiation exists that is defined only by a relatively higher number of cuticular ridges in the posterior part of the body for an equivalent length of the body. These ridges are more numerous in specimens from Corsica (*H. p. corsicus*) and North America (*H. p. bakeri*) than in those from Europe (*H. p. polygyrus*). This difference is also present in *H. p. polygyrus* and *H. p. bakeri* from China and Japan.

Unfortunately, the number of Japanese specimens, in Asakawa & Ohbayashi, (1986) (five males, five females) is too small to be conclusive unlike the work by Durette-Desset *et al.* (1972) where numerous specimens were observed. Therefore, we prefer at least temporarily, to identify the Chinese specimens as *H. neopolygyrus* without considering them as a subspecies.

## Discussion

*H. neopolygyrus* was described for the first time by Asakawa & Ohbayashi (1986) in *A. peninsulae* in the Abashiri area of Hokkaido Island (Japan). Asakawa *et al.* (1990) recorded *H. neopolygyrus* in *A. agrarius* from China. In this work, only the dorsal ray and the base of rays 8 of one specimen collected in Shenyang were illustrated and showed that the swelling at the base of rays 8 was absent; a feature which is characteristic of *H. neopolygyrus*. For this reason, Asakawa *et al.* (1990) identified their specimens as *H. neopolygyrus*. In the same article, the authors reported finding *H. neopolygyrus* in *A. agrarius* from Kyonggi-do (Korean peninsula) but provided no description or illustrations. Asakawa (1991) confirmed the presence of *H. neopolygyrus* in *A. agrarius* from China and the Korean peninsula as well as in *A. peninsulae* from Japan.

Asakawa *et al.* (1992) reported *H. polygyrus* in *A. uralensis* (= *A. microps*) from Ulumuchi (China). However, this was, in fact, a new species later named *H. asakawae* by Tenora & Barus (2001).

**Figs 17–23.**
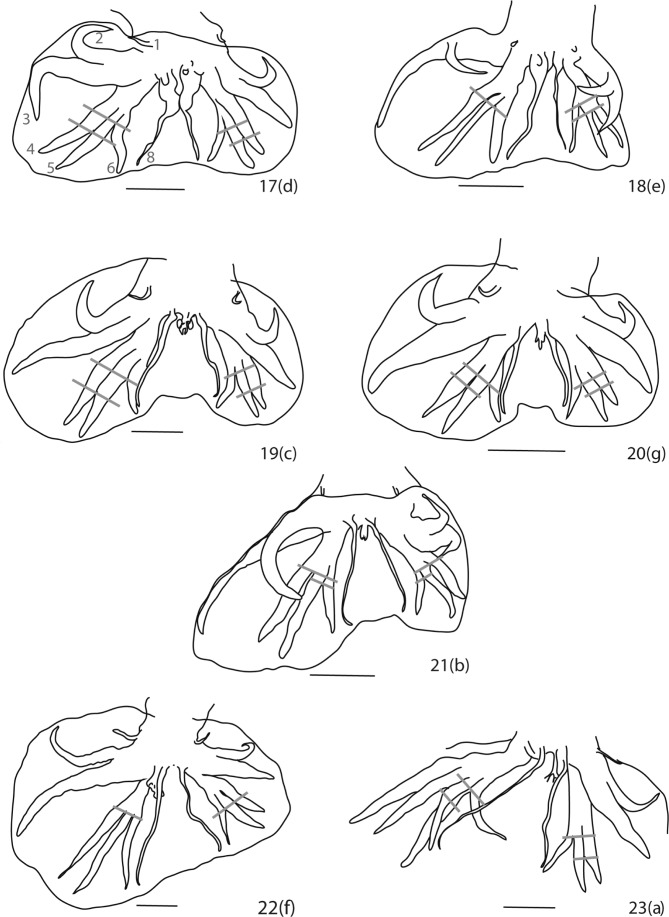
Caudal bursae of *Heligmosomoides* spp., from China, ventral views: 17, 18, *H. p. polygyrus* in *Mus musculus*, 17, from Shenyang, 18, from Changchun; 19, 20, *H. neopolygyrus* in *Apodemus agrarius*, 19, from Shenyang, 20, from Changsha; 21–23, *Heligmosomoides incertae sedis*, 21, in *A. agrarius*, from Antu, 22, in *A. peninsulae*, from Liang-Cheng, 23, in *A. peninsulae*, from Hulin. After Asakawa *et al*. (1993) and modified. Scale bar: Figs 17–23: 100 μm. Letters in brackets referred to Fig. 1 of Asakawa *et al*. (1993). Grey bars show level of divergence of rays 4 and 5 and arising of rays 6 on the common trunk of rays 4 to 6: Figs 17, 19, 20, 23, in both lobes rays 6 arise proximally to the level of divergence of rays 4 and 5; Fig. 18, in left lobe ray 6 arising proximally to the level of divergence of rays 4 and 5, in right lobe at same level as the divergence of rays 4 and 5; Fig. 21, in both lobes rays 6 arising slightly distally to the level of divergence of rays 4 and 5; Fig. 22, in both lobes, rays 6 arise at same level as the divergence of rays 4 and 5.

Asakawa *et al.* (1993) studied the distribution of *H. neopolygyrus* in the east of China in *A. agrarius* and in *A. peninsulae*. Moreover, they compared the morphological characteristics of rays 8 in *H. neopolygyrus* and in *H. p. polygyrus* from China. Although this article was in Japanese, the authors presented a map of eastern China, in which they included seven illustrations of caudal bursae in ventral view: two attributed to *H. p. polygyrus* (Figs 17–18) and five attributed to *H. neopolygyrus* (Figs 19–23) from the different provinces and different hosts. No measurements were provided. A detailed analysis of these caudal bursae using the known criteria and the new criteria provided in this study, allows us to conclude the recording of *H. neopolygyrus* in *A. peninsulae* by Asakawa *et al.* (1993) as erroneous. The five caudal bursae identified as *H. neopolygyrus* all clearly lacked the swelling at the base of rays 8 which is characteristic of *H. polygyrus* and differen-tiates it from *H. neopolygyrus*. However, these species can be distinguished from each other by the following features: (1) the relative distance between rays 6 and 8; and (2) the level at which rays 6 arise compared to the level of divergence of rays 4 and 5 on their common trunk. We consider that only the caudal bursae of the specimens parasitic in *A. agrarius* from Shenyang (Fig. 19) and Changsha (Fig. 20) may be identified as *H. neopolygyrus* due to the absence of swelling at the base of rays 8, with rays 6 arising proximally to the level of divergence of rays 4 and 5, and with rays 6 and 8 being parallel and close to each other.

In the other three specimens (Figs 21–23) rays 6 and 8 are distant from each other, which differentiates them from *H. neopolygyrus*. In addition, in the caudal bursa of the specimen from *A. agrarius* from Shenyang (Fig. 21), rays 6 arise just slightly distally to the level of divergence of rays 4 and 5 and in the one from Liang-Cheng (Fig. 22), rays 4–6 diverge at same level in both lobes. The specimen from Hulin (Fig. 23) is the only one with rays 6 arising proximally to the level of divergence of rays 4 and 5, as in *H. neopolygyrus*. In the absence of other features, particularly those of the synlophe, it is not possible to attribute a specific name to these species and we consider them as *Heligmosomoides incertae sedis* belonging to the “*polygyrus* line”. Considering as partially inaccurate identifications of Asakawa *et al.* (1993), we report for the first time the identification of *H. neopolygyrus* in *A. peninsulae* in Sichuan (central China).

**Fig. 24.**
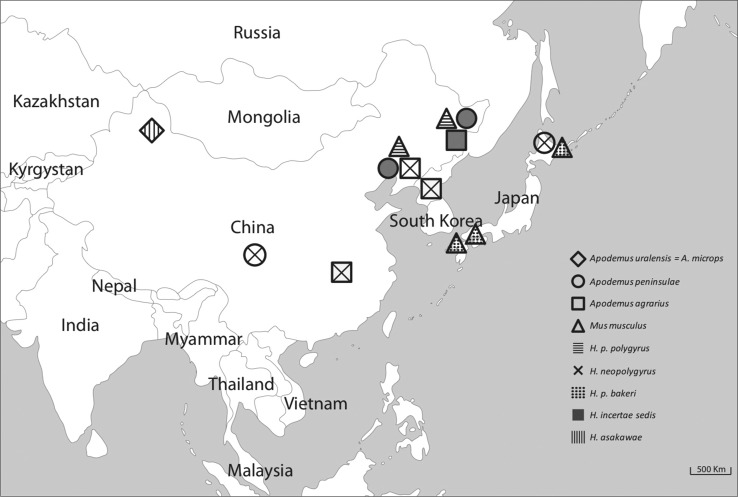
Distribution of the species of the genus *Heligmosomoides*, belonging to the “*polygyrus* line” modified from Asakawa (1988) from China and Japan.

Asakawa & Ohbayashi (1986) suggested that further studies may reveal the presence of *H. neopolygyrus* in *A. peninsulae* from the Northeast Palearctic region. This work supports a widespread distribution of *H. neopolygyrus sensu lato* which seems to follow its main host *A. peninsulae*. The presence of *H. neopolygyrus* in *A. agrarius* in China has been reported twice (Asakawa *et al.*, 1990, 1993) (Fig. 24).

Despite the discovery of new criteria to differentiate the species of the “*polygyrus*” complex reported in China and Japan, their systematic position remains uncertain due to incomplete descriptions and does not allow us to use certain potentially differentiating characters such as the number of cuticular ridges in the posterior part of the body. Both molecular and morphological studies need to be undertaken to determine their systematic rank (species or subspecies).
